# Targeted metabolome and transcriptome analyses reveal changes in gibberellin and related cell wall-acting enzyme-encoding genes during stipe elongation in *Flammulina filiformis*

**DOI:** 10.3389/fmicb.2023.1195709

**Published:** 2023-09-20

**Authors:** Hui Li, Sen Yao, Weiwei Xia, Xinbin Ma, Lei Shi, Huimin Ju, Ziyan Li, Yingli Zhong, Baogui Xie, Yongxin Tao

**Affiliations:** ^1^Institute of Cash Crops, Hebei Academy of Agriculture and Forestry Sciences, Shijiazhuang, Hebei, China; ^2^College of Horticulture, Fujian Agriculture and Forestry University, Fuzhou, Fujian, China; ^3^Mycological Research Center, College of Life Sciences, Fujian Agriculture and Forestry University, Fuzhou, Fujian, China

**Keywords:** *Flammulina filiformis*, gibberellin, stipe elongation, targeted metabolome, transcriptome

## Abstract

*Flammulina filiformis*, a typical agaric fungus, is a widely cultivated and consumed edible mushroom. Elongation of its stipe (as the main edible part) is closely related to its yield and commercial traits; however, the endogenous hormones during stipe elongation and their regulatory mechanisms are not well understood. Gibberellin (GA) plays an important role in the regulation of plant growth, but little has been reported in macro fungi. In this study, we first treated *F. filiformis* stipes in the young stage with PBZ (an inhibitor of GA) and found that PBZ significantly inhibited elongation of the stipe. Then, we performed GA-targeted metabolome and transcriptome analyses of the stipe at both the young and elongation stages. A total of 13 types of GAs were detected in *F. filiformis*; the contents of ten of them, namely, GA3, GA4, GA8, GA14, GA19, GA20, GA24, GA34, GA44, and GA53, were significantly decreased, and the contents of three (GA5, GA9, and GA29) were significantly increased during stipe elongation. Transcriptome analysis showed that the genes in the terpenoid backbone biosynthesis pathway showed varying expression patterns: *HMGS, HMGR, GPS*, and *FPPS* were significantly upregulated, while *CPS/KS* had no significant difference in transcript level during stipe elongation. In total, 37 *P450* genes were annotated to be involved in GA biosynthesis; eight of them were upregulated, twelve were downregulated, and the rest were not differentially expressed. In addition, four types of differentially expressed genes involved in stipe elongation were identified, including six signal transduction genes, five cell cycle-controlling genes, twelve cell wall-related enzymes and six transcription factors. The results identified the types and content of GAs and the expression patterns of their synthesis pathways during elongation in *F. filiformis* and revealed the molecular mechanisms by which GAs may affect the synthesis of cell wall components and the cell cycle of the stipe through the downstream action of cell wall-related enzymes, transcription factors, signal transduction and cell cycle control, thus regulating stipe elongation. This study is helpful for understanding the roles of GAs in stipe development in mushrooms and lays the foundation for the rational regulation of stipe length in agaric mushrooms during production.

## Introduction

*Flammulina filiformis* is a large-scale edible mushroom produced industrially worldwide for its excellent culinary and medicinal value, including antitumour, antihypercholesterolemic, antihypertension, and other therapeutic benefits (Rezaeian et al., 2021). The fruiting body of *F. filiformis* is mainly composed of the pileus and stipe, and the stipe is the main edible part (Tao et al., 2018). Due to the different cultivation conditions, the stipe of the artificially cultivated *F. filiformis* is much longer than that of the wild *F. filiformis*; and the elongation of the stipe directly affects the value of the product. Therefore, it is of great interest to study the growth and developmental patterns of stipe elongation in *F. filiformis*. The stipe elongation is a complex biological process that may be regulated by a variety of intracellular hormones and signals. Fungal cytoplasm is enclosed in the cell wall which is able to expand longitudinally driven by cell expansion pressure (Liu et al., 2021). The expansion and elongation of the stipe cell wall depend on the action of cell wall hydrolases (Cabib et al., 2001; Klis et al., 2002). The two major components, namely chitin and β-glucan, constitute the polysaccharide network structure of the fungal cell wall. During the stipe elongation process, chitinase and glucanase jointly participate in inducing the elongation of the cell wall, so they play a key role in the stipe elongation (Zhang et al., 2014; Niu et al., 2015; Liu et al., 2021). Li et al. identified three exo-β-1,3-glucanases, one β-1,6-glucan synthase, four chitinases, and two expansin proteins, and the transcript levels of these genes were closely associated with stipe elongation in *F. filiformis* (Li et al., 2022). In *Agaricus bisporus*, β-1,6-glucan synthase gene expression was upregulated in mature fruiting bodies, and β-1,6-linked glucan side branches were also more numerous in rapidly elongating cell walls, suggesting that β-1,6-glucan synthase may be involved in changes in cell wall composition (Mol and Wessels, 1990). The elongation and growth of the stipe cell wall were found to be mainly due to the hydrolytic enzymes that can hydrolyse the polysaccharide matrix on the cell wall and promote the elongation and growth of the stipe by continuously disrupting and reconstructing the cross-linked structure between glucan and chitin, thus maintaining the plasticity of the cell wall in *Coprinopsis cinerea* (Kamada et al., 1985, 1991; Kang et al., 2019; Bai et al., 2020; Liu et al., 2020). However, little is known about how these genes and the upstream hormones are regulated during the stipe elongation.

In plants, the main growth-regulating hormones are gibberellins (GAs). Their main physiological function in plants is to promote plant growth and development, mainly through an increase in cell number and cell elongation. GA can increase cell division in some plants (e.g., *Hyoscyamus niger L*.), and it can promote cell division because it shortens the time between the G1 and S phases of the cell cycle (Yang and Xiao, 2004). The biosynthetic pathway of GA in plants is divided into three steps according to the characteristics of the synthesizing enzyme (Tan and Ma, 2008): (i) the pathway of GGPP formation, a precursor of GA synthesis; (ii) GA12-7-aldehyde synthesis; and (iii) synthesis of other GAs from GA12-7-aldehyde. Editing of GA synthesis genes can accelerate and retard the plant growth process. Xiao et al. used RNA interference to silence three enzyme-encoding genes in the tomato GA20-oxidase family to obtain dwarf tomato plants (Xiao et al., 2006). GA not only promotes the elongation growth of plants but also has a significant promotion effect on the growth and development of fungi (El-Bahrawi, 1982; Wu et al., 2021).

GAs are diterpenoids (Kawaide, 2006), and 136 GA species have been found in plants, fungi, and bacteria, most of which are found in higher plants and some in fungi (Tan and Ma, 2008). The mevalonate (MVA) pathway is a biosynthetic pathway for fungal terpenoids (Lu et al., 2014; Ali et al., 2017), which is a part of terpenoid backend biosynthesis. HMG-CoA synthase (HMGS) is a key enzyme in the MVA pathway, and its role is to catalyze the condensation of acetoacetyl-CoA to HMG-CoA together with acetyl-CoA acetyltransferase (AACT; Bach et al., 1991). The main function of HMG-CoA reductase (HMGR) is to catalyze the irreversible production of MVA from HMG-CoA, and it is the first important rate-limiting enzyme in the MVA pathway (Bach, 1986; Choi and Bostock, 1992). GPS catalyzes 1 molecule of IPP with DMAPP to form GPP, and FPPS catalyzes 2 molecules of IPP to condense with DMAPP to form FPP. GGPPS catalyzes 3 molecules of IPP with DMAPP to form GGPP, providing a C20 backbone for diterpene formation (Ma et al., 2006). Liu et al. used metabolome and transcriptome to jointly analyze *Sanghuangporus baumii* at different developmental stages and found that GA4 acted in the protoplast and cotyledon stages. The highest GA4 content was found in the primordium stage; the *IDI* gene promoted the transcription of the GGPS gene leading to an increase in GA4 content, which, in turn, promoted the growth of the fruiting body (Liu et al., 2022b). The pathway of GA synthesis in fungi is well-studied in *G. fujikuroi* and consists of three main stages (Kawaide, 2006; Yamaguchi, 2008): the synthesis of GGPP (geranylgeranyl diphosphate, GGPP), the synthesis of GA12 aldehyde from GGPP, and the conversion of GA12 aldehyde to other species of GA. Intermediate metabolites in steps 1 and 2 are present in both plants and fungi, and it is noteworthy that the synthesis of *ent*-kaurene in plants is catalyzed by two enzymes, namely CPS and KS, whereas the synthesis of *ent*-kaurene from *G. fujikuroi* is directly catalyzed by a single CPS/KS bifunctional enzyme that catalyzes GGPP (Kawaide, 2006). The process of synthesizing other GAs from GA12-7-aldehyde in step 3 is distinctly different in plants and fungi because of different enzymes and enzymatic substrates that act on the corresponding products (Liu et al., 2022a).

The role of the GA synthesis pathway in stipe development in *F. filiformis* has not been reported, and its detailed functions and regulatory mechanisms in *F. filiformis* are not yet clear. In the present study, we first identified the type and content of GAs by GA-targeted metabolome and detected the expression patterns of genes related to the synthesis pathway during the stipe elongation by RNA-Seq. These results will help us to better understand the changes in GA and a series of related genes during the stipe elongation.

## Materials and methods

### Strains and culture conditions

The yellow *F. filiformis* strain F006 was conserved at the Institute of Cash Crops, Hebei Academy of Agriculture and Forestry Sciences. The strain was activated in potato dextrose agar (PDA) medium and incubated at 25°C at constant temperature and protected from light; substrate cultivation experiments were conducted according to the method described by Tao et al. (2019), and some modifications were made. The cultivation medium was composed of 52.5% cottonseed shells, 15% sawdust, 25% wheat bran, 5% corn powder, 2% calcium sulfate dehydrate, and 0.5% pulverized lime in 60% water. The cultivation bottles containing 200 g sterile medium were inoculated with six equipotent PDA blocks with mycelia and placed in an incubator at 23°C for mycelial growth (20 days). After the mycelia filled the bottles, the bottles were moved to an incubator at 10°C and 90% humidity to promote primordium formation and fruiting body development. The samples were collected on the 4th day (young fruiting body stage) and the 7th day (elongation stage) of the stipe, and the top 1 cm of the stipe was taken after removing the pileus. The samples were wrapped in tin foil and placed into liquid nitrogen immediately after sampling and then frozen at −80°C for transcriptome sequencing and GA content determination.

Paclobutrazol (PBZ, the working concentration is 200 mg/L) was sprayed on young fruiting bodies as an inhibitor of GA synthesis (Duan et al., 2019). The treatments were applied twice a day at 12 h intervals for 3 days. The stipe length was measured at the end of the treatments, and there were three biological replicates.

### Sample preparation and LC-MS/MS analysis for the determination of GA content

Hormone extraction: (1) take out the ultra-low temperature preserved sample, grind it in liquid nitrogen to dry powder, weigh the appropriate amount of fresh sample in a glass test tube; (2) add isopropyl alcohol-water-hydrochloric acid extraction solution to the glass test tube; (3) add 8 μL of 1 μg/ml internal standard solution and shake at low temperature for 30 min; (4) add dichloromethane and shake at low temperature for 30 min; (5) centrifuge at low temperature for 5 min at 13,000 r/min and extract the lower organic phase; (6) avoid light, blow dry the organic phase with nitrogen, and re-solvate with methanol (0.1% formic acid); (7) centrifuge at 4°C for 10 min (13,000 *g*) and pass the supernatant through a 0.22 μm filter membrane, then carry out HPLC-MS/MS detection (the extraction process should be kept on the ice box at 4°C).

Liquid phase conditions: (1) column: Poroshell 120 SB-C18 reversed-phase column (2.1×150, 2.7 μm); (2) column temperature: 30°C; (3) mobile phase: A:B=(methanol/0.1% formic acid):(water/0.1% formic acid); (4) elution gradient: 0–1 min, A = 20%; 1–9 min, A increasing to 80%; 9–10 min, A = 80%; 10–10.1 min, A decreasing to 20%; 10.1–15 min, A = 20%; (5) injection volume: 2 μl, A = 80%; 9–10 min, A = 80%; 10–10.1 min, A decreases to 20%; 10.1–15 min, A = 20%; (5) Injection volume: 2 μl.

Mass spectrometry conditions: electrospray ionization (ESI) source atomization temperature: 400°C, curtain gas (CUR): 15 psi, spray voltage, IonSpray voltage (IS): 4,500 V, atomization gas pressure (Gas1): 65 psi, auxiliary gas pressure (Gas2): 70 psi, monitoring mode: multiple reaction monitoring mode (MRM). Gas2: 70 psi, monitoring mode: MRM (multiple reaction monitoring mode). In the Q-Trap 6500, each ion pair is scanned for detection based on optimized declustering potential (DP) and collision energy (CE). The amount of GA is quantified in ng/g.

### RNA-seq and DEG analysis

Samples used for RNA extraction weighed 0.5 g. Total RNA was extracted from samples using the OMEGA E.Z.N.A. A plant RNA kit (R6827-01; Omega Bio-Tek, USA) was used for transcriptome sequencing when the A260/A280 ratio of RNA was between 2.0 and 2.2 (measured using Thermo Scientific NanoDrop™ Lite; Wilmington, DE, USA).

The extracted RNA was entrusted to high-throughput transcriptome sequencing by Beijing Novogene Technology Co., Ltd. Transcriptome data preprocessing is as follows: First, quality assessment of the raw transcriptome data was performed using FastQC, followed by quality control using fastap software to obtain clean data. The SAM file was obtained by comparing the clean data using Hisat2 software using the reference genome and annotation information of the laboratory-assembled *F. filiformis* (6-3). Subsequently, after converting the SAM files to BAM files and reordering them using SAMtools software, the raw read counts of each gene in each sample were obtained using FeatureCounts software, and after filtering out the non-expressed genes using awk command, the count values were then converted to FPKM values using TBtools (Chen et al., 2020). In this study, R software was used to obtain the normalized gene expression of RNA-Seq data for subsequent analysis. Differentially expressed genes (DEGs) were obtained by screening with difference multiplicity |log2FC|>1 and *P* < 0.05, based on transcriptome sequencing preprocessing results. The obtained DEGs were enriched using the KEGG enrichment, GO enrichment, and KOG enrichment analysis tools of the GENE DENOVO Cloud platform (http://www.omicshare.com/tools/).

### Statistical analysis

The values in the statistical plots are the mean ± standard deviation of the three biological replicates; the significance analysis was performed using a *t*-test. *P*-values of < 0.05 were considered to be statistically significant.

## Results

### Stipe development process of the *F. filiformis* strain FY006

**Figure 1:**
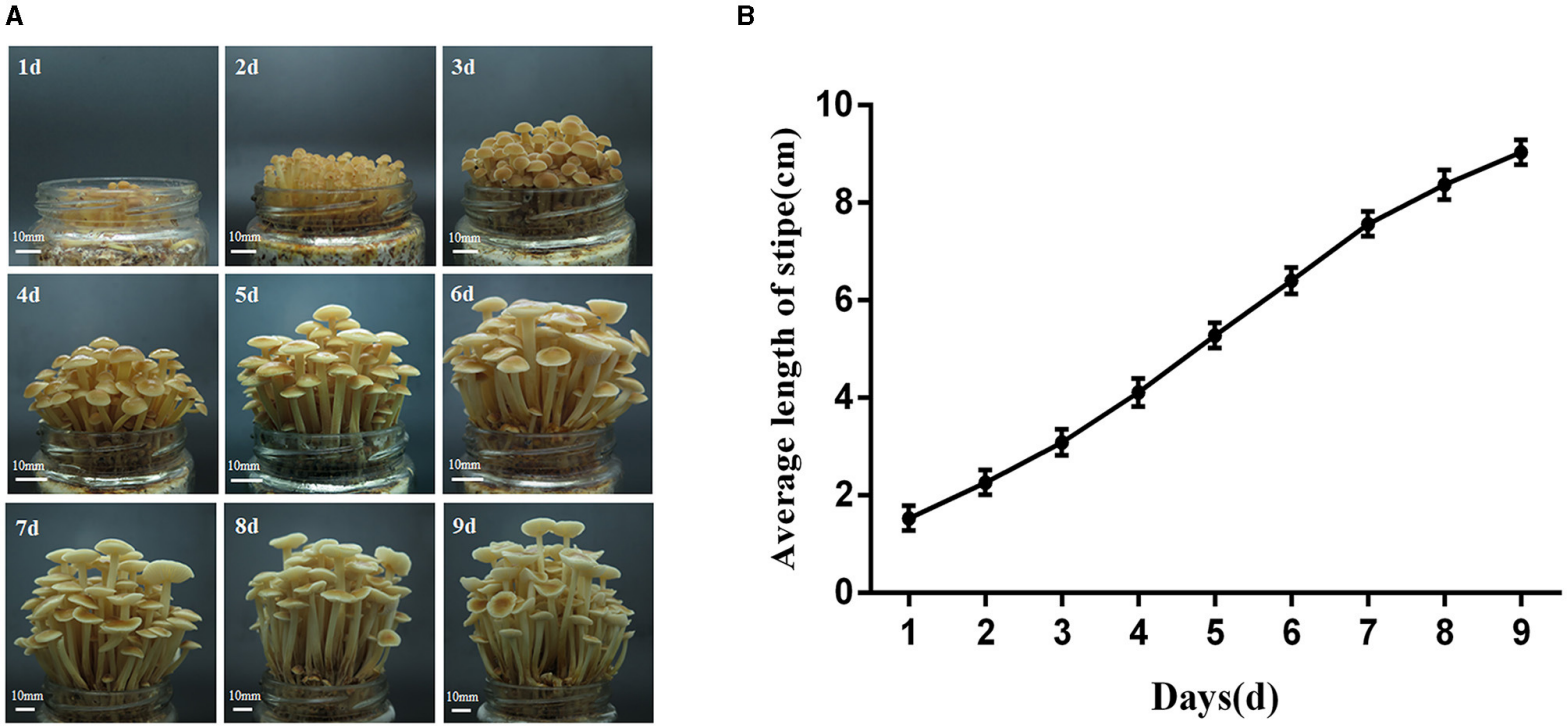
Elongation dynamics of the stipe of the F006 after growing out of the primordium. **(A)** The growth phenotype of the fruiting body of *F. filiformis* (bar = 10 mm). **(B)** Variation in the length of the stipe of *F. filiformis*.

After growing out of the primordium, the growth of the fruiting body of *F. filiformis* strain FY006 is shown in Figure 1A, and the dynamics of the stipe elongation is shown in Figure 1B. The growth rate of the stipe is fastest on the 4th to 7th days after it grew out of the primordium, so the fruiting body on the 4th (young fruiting body stage, YF) and 7th (elongation stage, EL) days is collected, and the pileus is removed. After that, 1 cm of the tip of the stipe is taken for GA content measurement and transcriptome sequencing.

### Inhibition of stipe elongation in *F. filiformis* by PBZ

**Figure 2:**
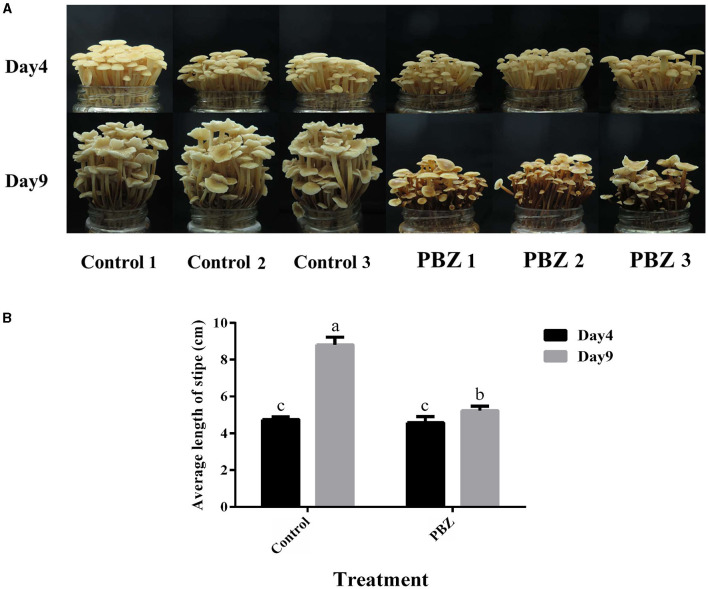
Effect of PBZ treatment on the growth of *F. filiformis* fruiting body. **(A)** Growth phenotype of fruiting body before and after treatment (bar = 10 mm). **(B)** Stipe length before and after treatment. Different lowercase letters indicate significant differences at *p* < 0.05.

The effect of PBZ (an inhibitor of GA biosynthesis) on the growth of *F. filiformis* was investigated, and the phenotypes of the fruiting bodies before and after PBZ treatment are shown in Figure 2A. The length of the stipe of each treatment group is shown in Figure 2B. After spraying PBZ, the stipe length was significantly lower than that of the control group, and the inhibition rate reached 83.72%. We can conclude that the GA inhibitor PBZ inhibits the stipe elongation of *F. filiformis*.

### GA targeted metabolome during the stipe elongation

**Figure 3:**
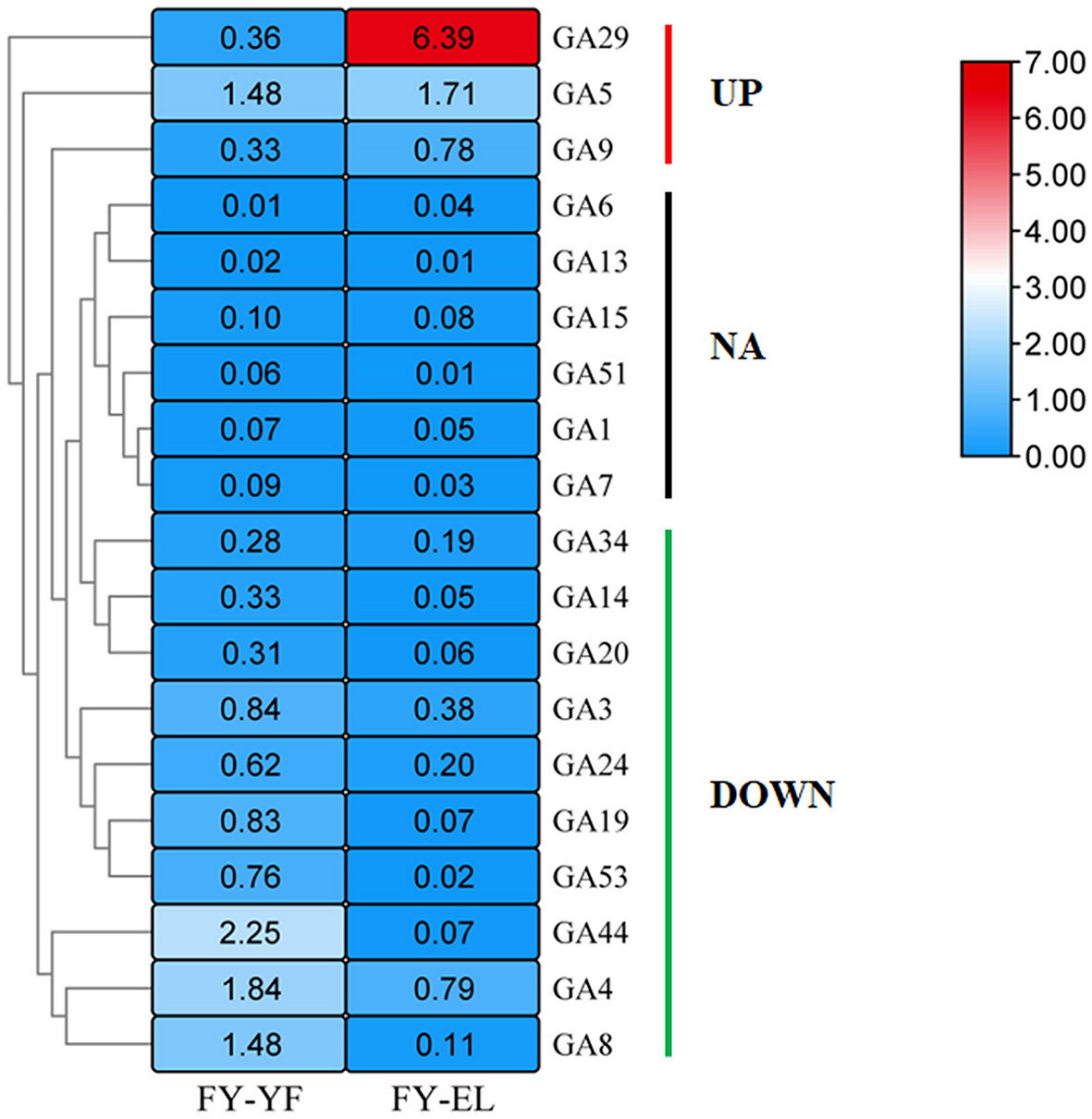
Heatmap of gibberellin content (ng/g) in the young fruiting body stage and elongation stage of *F. filiformis* stipe.

To investigate the synthesis and accumulation of GAs in the stipe of *F. filiformis*, targeted metabolomic analysis was performed on stipe samples at different developmental stages, and the contents of 19 GAs in the stipe of *F. filiformis* at the young fruiting body stage and elongation stage were determined by LC-MS/MS, namely GA1, GA3, GA4, GA5, GA6, GA7, GA8, GA9, GA13, GA14, GA15, GA19, GA20, GA24, G29, GA34, GA44, GA51, and GA53; the heatmap of GA content (mean values of three biological replicates) is shown in Figure 3. The detection limit of GA was 0.1 ng/g. When the analyte was ≤0.1 ng/g in both stages, we marked the result as non-detected (NA) according to the detection limit, the GA species marked as NA included GA1, GA6, GA7, GA13, GA15, and GA51, a total of six species, and we conducted the next study on the remaining 13 species of GAs.

Among the 13 GA species with detectable levels, the highest GA29 content was found in the elongation stage stipe. Ten species of GAs were downregulated from the young fruiting body to the elongation stipe, namely, GA3, GA4, GA8, GA14, GA19, GA20, GA24, GA34, GA44, and GA53, among which nine species were downregulated by more than 50% except for GA34, which was downregulated by 33.6%. Among GAs with increased content were GA5, GA9 and GA29, which were present at significantly higher levels during the elongation stage than during the young fruiting body stage, with GA5 increasing by 15.5% and GA9 and GA29 increasing by more than 2-fold.

**Figure 4:**
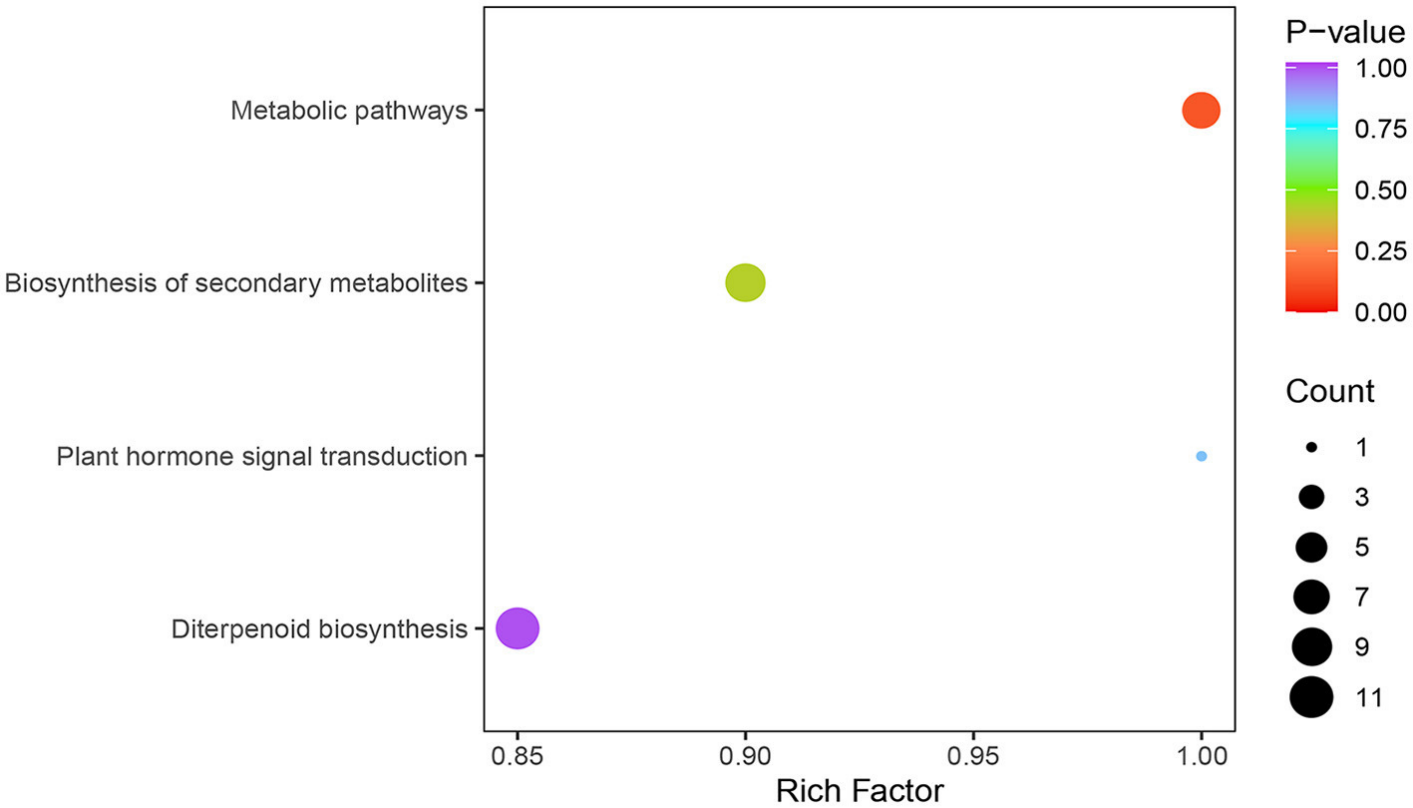
KEGG enrichment analysis of differentially metabolized species of gibberellins.

There are four main metabolic pathways analyzed by KEGG enrichment of GA differential metabolic species in the young fruiting body stage and elongation stage of *F. filiformis* stipe (Figure 4): ko00904: diterpenoid biosynthesis, which includes GA biosynthesis; ko01100: metabolic pathways; ko01110: biosynthesis of secondary metabolites; and ko04075: plant hormone signal transduction.

### Enrichment analysis of differentially expressed genes

To investigate the molecular regulation mechanism of GA during the development of *F. filiformis* stipe, transcriptome sequencing was performed on two developmental stages of the stipe. The six sequenced samples yielded 41.6 G and 277,263,274 raw reads and 41.04 G and 273,533,810 clean reads after filtering. The GC content ranged from 53.07 to 53.31%, and the Q30 ranged from 91.87 to 92.96%. This indicates that the sequencing results of the samples were credible, and the genes with significant expression differences were screened for subsequent analysis. There were 255 upregulated DEGs and 380 downregulated DEGs in the elongation stage (EL) compared with the young fruiting body stage (YF), for a total of 635 DEGs.

**Figure 5:**
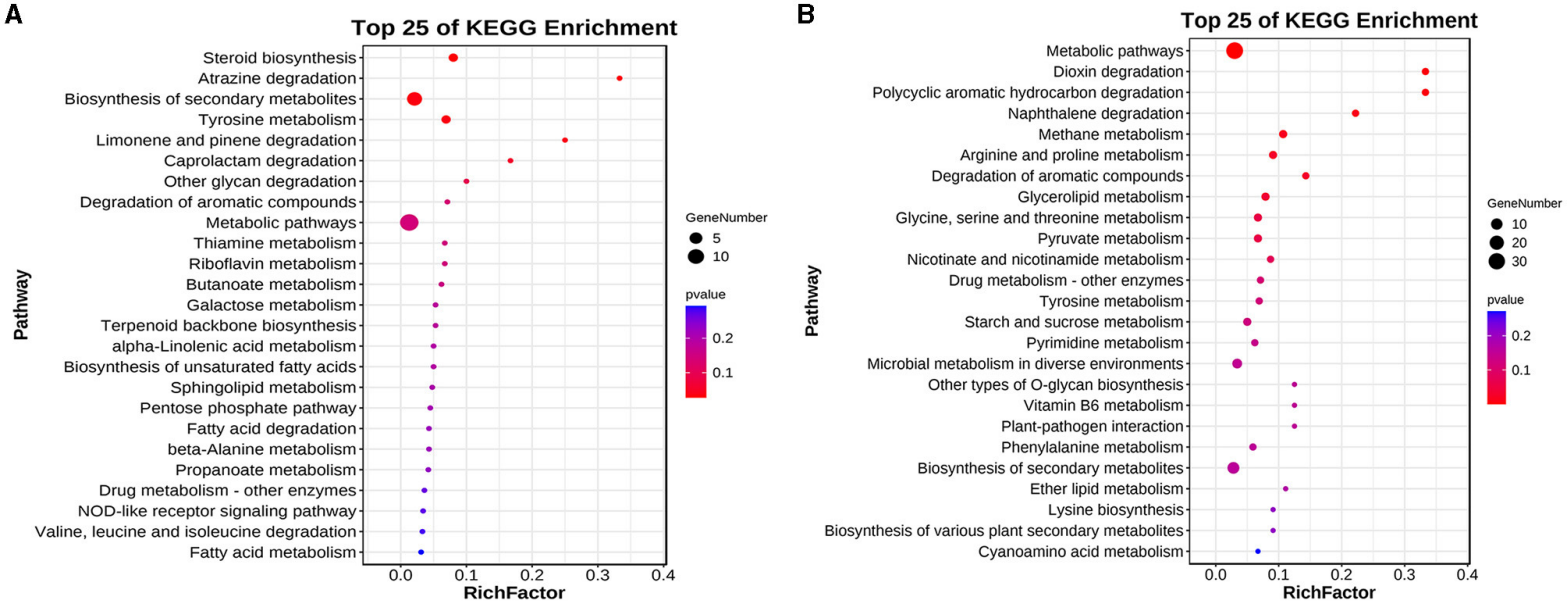
Differential genes KEGG enrichment analysis. **(A)** Enrichment results of upregulated genes. **(B)** Enrichment results of downregulated genes.

The results of the KEGG enrichment analysis of differentially expressed genes showed that the DEGs upregulated in the elongation stage were mainly enriched in lipid metabolism, carbohydrate metabolism, glycan biosynthesis and metabolism, biosynthesis of secondary metabolites, and terpenoid backbone biosynthesis (Figure 5A). The downregulated DEGs in the elongation stage were mainly enriched in amino acid metabolism, glycan biosynthesis and metabolism, metabolism of cofactors and vitamins, pyrimidine metabolism, and starch and sucrose metabolism (Figure 5B). However, no differential genes were found in diterpenoid biosynthesis, which is inconsistent with the previous results of metabolite analysis. This inconsistency may be because genes related to the diterpenoid biosynthesis pathway in *F. filiformis*, which includes the GA biosynthesis pathway, were not annotated in the KEGG background database.

A GO enrichment analysis of differentially expressed genes upregulated in the stalk of *A. chinensis* from the juvenile stage to the mature stage showed that cellular components (CC) were mainly enriched in membrane (GO:0016020), endoplasmic reticulum (GO:0005783), transporter complex (GO:1990351), and oxidoreductase complex (GO:1990204); molecular function (MF) was mainly enriched in oxidoreductase activity (GO:0016491), transmembrane transporter activity (GO: 0022857), gluconokinase activity (GO:0046316), and transcription regulatory region sequence-specific DNA binding (GO:0000976); biological process (BP) is mainly enriched in response to stimulus (GO:0050896), oxidation–reduction process (GO:0055114), secondary alcohol biosynthetic process (GO:1902653), and oligopeptide transport (GO:0006857; Figure 6A). A GO enrichment analysis of the downregulated differentially expressed genes showed that the cellular component (CC) was mainly enriched in mitochondrial part (GO:0044429), fungal-type cell wall (GO:0009277), and protein farnesyltransferase complex (GO:0005965); molecular function (MF) is mainly enriched in transporter activity (GO:0005215), hydrolase activity, hydrolyzing O-glycosyl compounds (GO:0004553), and glycerol kinase activity (GO:0004553); biological process (BP) was mainly enriched in polysaccharide catabolic process (GO:0000272), carbohydrate transmembrane transport (GO:0034219), polyamine metabolic process (GO:0006595), and protein farnesylation (GO:0018343; Figure 6B).

**Figure 6:**
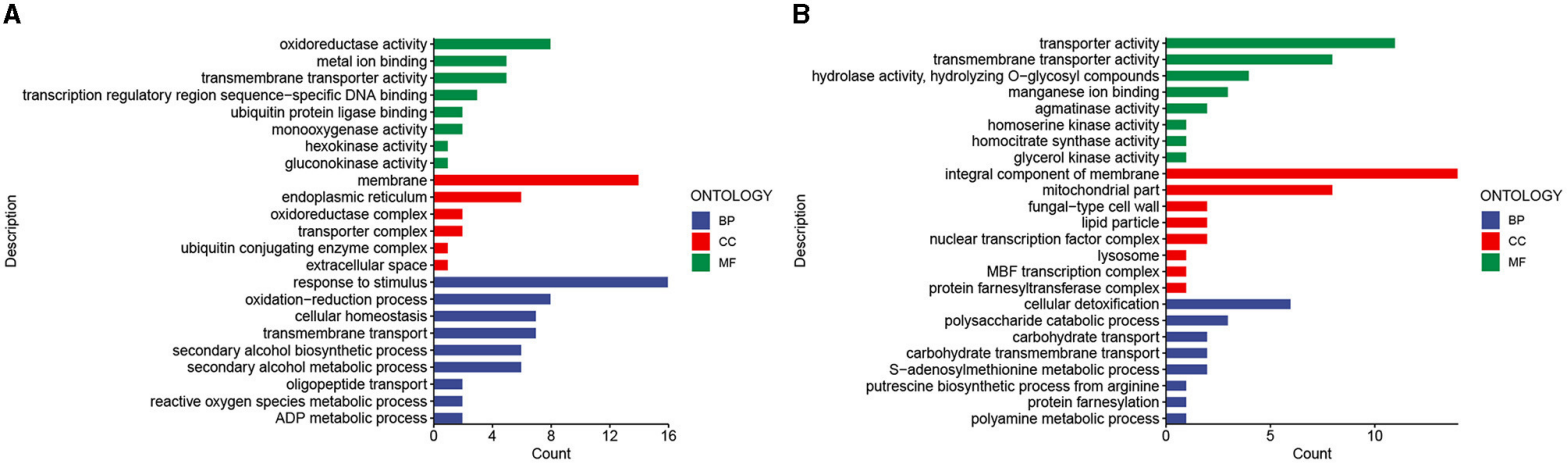
Differential gene GO enrichment analysis. **(A)** Enrichment results of upregulated genes. **(B)** Enrichment results of downregulated genes.

To further analyze the function of DEGs, KOG functional classification of differential genes was conducted, with 17 groups. There were 13 common groups of upregulated and downregulated genes, namely, E, G, I, K, L, O, P, Q, R, S, T, U, and V. The groups with higher concentrations of upregulated and downregulated genes include O, G, I, Q, and R. The specific groups of upregulated genes include B (chromatin structure and dynamics), J (translation, ribosomal structure, and biology), M (cell wall/membrane/envelope biology), and Z (cytoskeleton). The specific groups of downregulated genes include A (RNA processing and modification), C (energy production and conversion), F (nucleotide transport and metabolism), and Y (nuclear structure). Among the specific groups, the C group enriched with downregulated genes had the largest difference.

### Identification of the genes of the GA biosynthesis pathway and their expression patterns during stipe elongation

**Figure 7:**
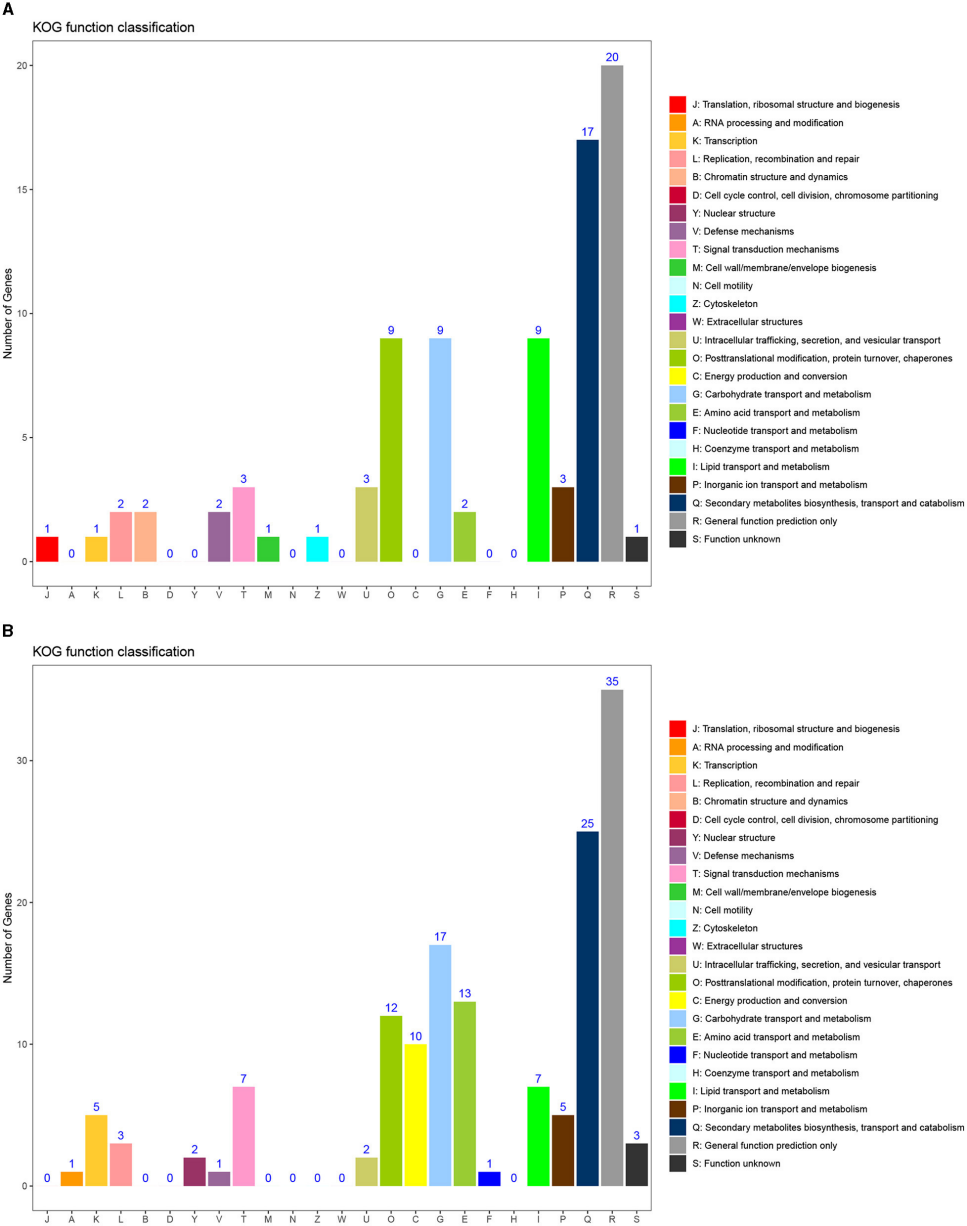
KOG functional classification map of differential genes. **(A)** KOG enrichment results of upregulated genes. **(B)** KOG enrichment results of downregulated genes.

**Figure 8:**
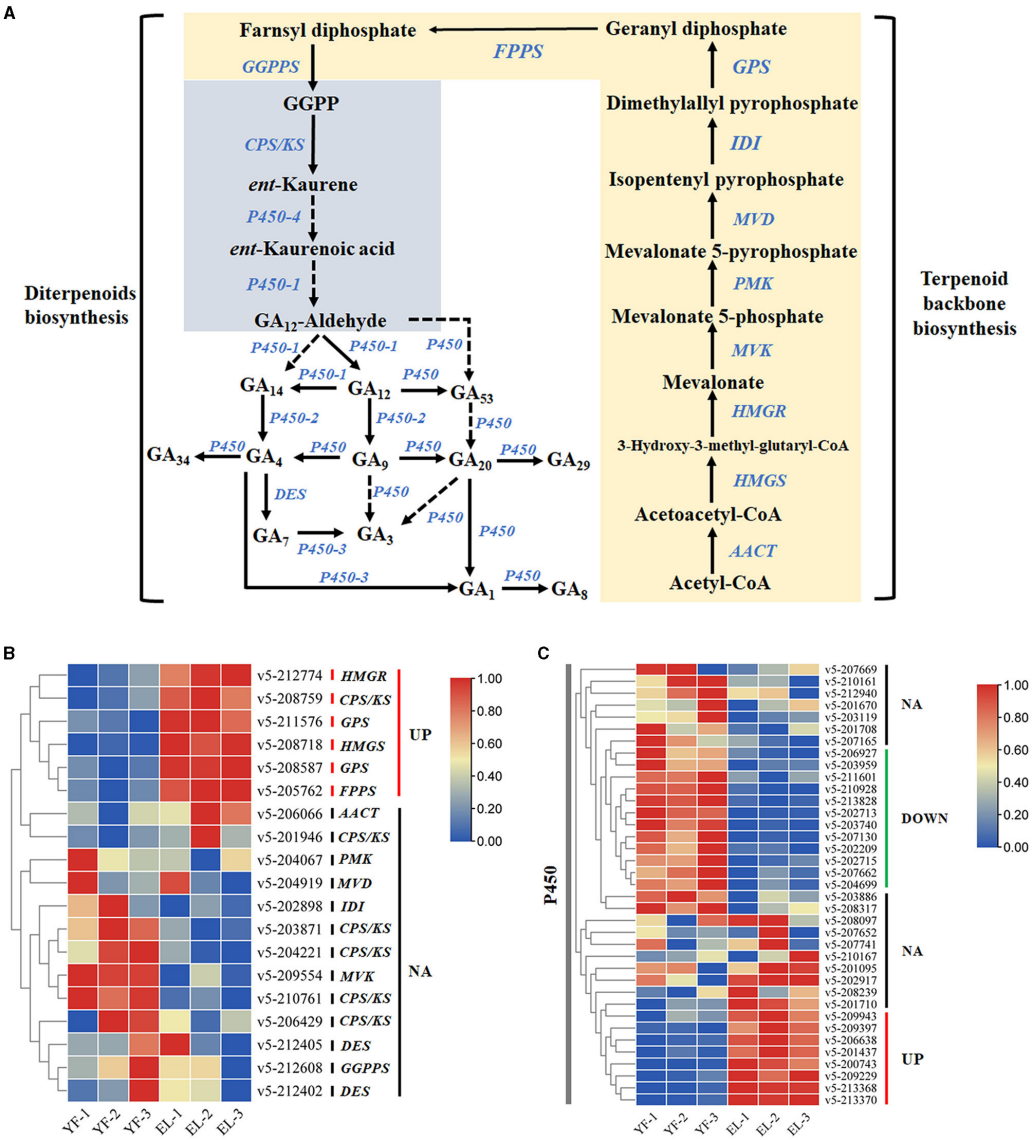
Identification of genes related to the gibberellin synthesis pathway. **(A)** Terpenoid backbone and gibberellin biosynthesis pathway. **(B)** Differential expression of DEGs associated with gibberellin biosynthesis during the stipe elongation of *F. filiformis*. **(C)** Differential expression of DEGs associated with P450 during the stipe elongation of *F. filiformis*.

Using genes related to terpenoid backbone biosynthesis and diterpenoid biosynthesis (contains GA biosynthesis), the KEGG pathway related to terpenoid biosynthesis in plants and fungi, as a reference, we used local BLASTp with 6-3 as the reference genome, setting an e-value<1e-5, and combined the results with the annotation results of the three major databases SwissProt, TrEMBL, and Nr to obtain homologues that might be involved in GA biosynthesis in *F. filiformis* (Figure 7). Eleven genes were annotated to terpenoid backbone biosynthesis in *F. filiformis*, including one each of *AACT, HMGS, HMGR, MVK, PMK, MVD, IDI, FPPS*, and *GGPPS* and two *GPS*; 45 genes were annotated to diterpenoid biosynthesis including six *CPS/KS*, 37 *P450*, and 2 *DES*. Combined with the results of transcriptome analysis, five genes were screened for differential expression during terpenoid backbone biosynthesis (Figure 8); *HMGS, HMGR*, 2 *GPS*, and *FPPS* were significantly upregulated. In total, one *CPS/KS* and eight annotated *P450* genes were upregulated in expression during diterpenoid biosynthesis, 12 genes were downregulated in expression, and 17 genes were not differentially expressed. *HMGS, HMGR, GPS*, and *FPPS* are key enzymes for the synthesis of GGPP (a common precursor of diterpenoid synthesis), and all four enzyme-encoding genes were upregulated in expression, which would increase the formation of the diterpenoid synthesis precursor GGPP and thereby affecting the synthesis of GA.

### Four important types of DEGs involved in the stipe elongation

**Figure 9:**
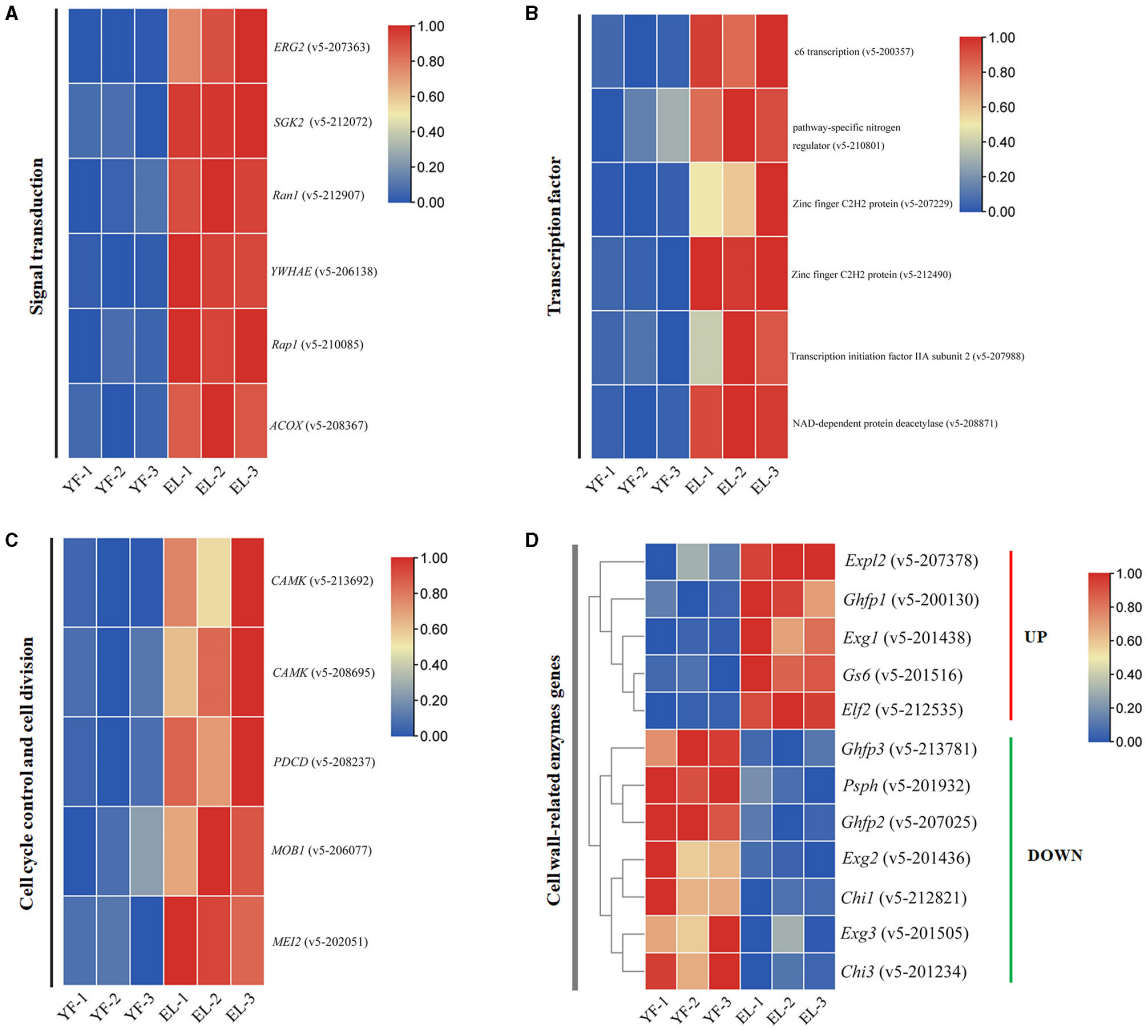
Four important types of DEGs involved in the stipe elongation. **(A)** Signal transduction pathways involved in the stipe elongation process of *F. filiformis*. **(B)** Differential expression of transcription factor during the stipe elongation of *F. filiformis*. **(C)** Cell cycle control pathway involved in the stipe elongation process of *F. filiformis*. **(D)** Differential expression of cell wall-related enzyme genes during the stipe elongation of *F. filiformis*.

The signal transduction pathway is an important pathway involved in the growth and development of *F. filiformis* in response to endogenous hormones. Six upregulated DEGs involved in the signal transduction pathway were screened using the KEGG pathway and KOG enrichment results combined with transcriptome data (Figure 9A), including the cAMP signaling pathway (v5-208367), MAPK signaling pathway (v5-206138), Rap1 signaling pathway (v5-210085), and KOG enrichment to T class (signal transduction mechanisms), with three upregulated genes v5-207363 (C-8 sterol isomerase), v5-212072 (FoxO signaling pathway, PI3K-Akt signaling pathway), and v5-212907 (Ran1-like protein kinase).

Signaling pathways are usually directed to transcription factors for global regulation. The differentially expressed transcription factor genes were also identified during the stipe elongation. Six upregulated transcription factors from the young stage to the elongation stage were identified based on the results of enrichment analysis (Figure 9B). It mainly includes c6 transcription (v5-200357), pathway-specific nitrogen regulator (v5-210801), zinc-finger C2H2 protein (v5-207229), zinc-finger C2H2 protein (v5-212490), transcription initiation factor IIA subunit 2 (v5-207988), and NAD-dependent protein deacetylase (v5-208871).

Cell cycle control and cell division are also important processes involved in stipe elongation. Using the KEGG pathway associated with cell cycle control and cell division, five upregulated DEGs involved in cell cycle regulation were identified in combination with transcriptomic data (Figure 9C). The main proteins involved in the following pathways were CAMK/CAMKL/GIN4 protein kinase (v5-213692, v5-208695), Programmed cell death protein (v5-208237), MOB kinase activator (v5-206077), and Protein MEI2-like 2 (v5-202051).

According to the results by Li et al., the rapid elongation of the stipe is also closely related to the remodeling of the cell wall structure. We investigated the transcription level of cell wall-related enzyme-encoding genes in *F. filiformis* during stipe elongation. A heatmap of the differential gene expression of cell wall-related enzymes based on the RNA-Seq results, as shown in Figure 9D, showed that the expression of *Exg1* gene of exo-β-1,3-glucanase was significantly upregulated; the expression of *Exg2* and *Exg3* genes was significantly downregulated; the expression of the *Gs6* gene, encoding β-1,6-glucan synthase, was significantly upregulated; the expression of the *Chi1* and *Chi3* chitinase genes was significantly downregulated; *Ghfp1* (glycoside hydrolase family protein) was significantly upregulated and *Ghfp2* and *Ghfp3* were significantly downregulated; *Elf2* (elongation factor 2) gene was significantly upregulated; *Psph* (phosphopyruvate hydratase) gene was significantly downregulated.

## Discussion

The elongation of the stipe is an important biological problem for *F. filiformis* and all agaric fungi. In this study, we applied targeted metabolomic and transcriptomic analyses to reveal the molecular mechanism by which GA regulates stipe elongation. First, we determined the contents of 19 GAs in the stipe of *F. filiformis* using LC-MS. Among the 13 detectable GAs, 10 GAs (GA3, GA4, GA8, GA14, GA19, GA20, GA24, GA34, GA44, and GA53) were downregulated during the elongation stage, and 3 GAs (GA5, GA9, and GA29) were upregulated during the elongation stage. The changes in GA content may be closely related to the elongation of the stipe, and the accumulation of various GAs in the young fruiting body stage may lay the foundation for the rapid elongation of the stipe in the following developmental period. Among more than 130 GAs identified so far, GA1, GA3, GA4, and GA7 had the highest biological activity in plant (Yamaguchi, 2008), while the fungi *G. fujikuroi, Phaeosphaeria* sp. L487, *Sphaceloma*, and *Neurospora crassa* produce and accumulate GA3 and/or GA4 as the final GA metabolites; GA4 has also been detected in *Sanghuangporus baumii* which is a medicinal fungus (Liu et al., 2022b). The final GA metabolite of *Phaeosphaeria* sp.L487 was GA1, and further analysis of its GA metabolites included GA12, GA15, GA20, GA24, GA25, and GA82 (Sassa et al., 1994; Kawaide et al., 1995; Seto, 1995). However, GA14, a key intermediate of GA metabolism in *G. fujikuroi* and *Sphaceloma* sp., was not detected, in contrast to the GA species detected in *F. filiformis*.

GA, as a growth regulator, has more applications and concerns in plant growth and development than in fungi, but it is also closely related to the growth and development of fungi (Hedden et al., 2001; Tudzynski, 2005). In *F. filiformis*, the content of most species of GAs (including GA4) was downregulated with the elongation of the stipe, and similarly, the GA4 content showed a downregulated pattern from the primordium stage to the fruiting body stage in *Sanghuangporus baumii*, with a similar trend of change in *F. filiformis* (Liu et al., 2022b). The expression of HMGS and HMGR genes was upregulated during the stipe elongation of *F. filiformis*, which is consistent with the increasing expression pattern of *HMGS* and *HMGR* genes in *Sanghuangporus baumii* with growth and development. GGPP is a synthetic precursor of GA (Hedden and Phillips, 2000). This may be due to the large consumption and utilization of the precursor GGPP that the total amount of GGPP precursors is insufficient, which in turn, feeds back to regulate the terpenoid backbone biosynthesis pathway, and the expression of related genes is upregulated which, in turn, promotes the synthesis of GGPP. In this study, 37 *P450* genes in *F. filiformis* that may be involved in GA biosynthesis were identified based on gene homology comparisons reported in *Sanghuangporus baumii*, of which expression of eight genes were upregulated, expression of 12 genes were downregulated, and expression of 17 genes were without differences. The specific functions of these *P450* genes could not be clarified due to the extremely high similarity of the conserved domains of *P450* genes. P450 genes in fungi and plants have similar catalytic activities, but their amino acid sequences are very different (Keller and Hohn, 1997).

The signal transduction pathway is an important pathway involved in growth and development of *F. filiformis* in response to endogenous hormones. Studies have shown that heterotrimeric G proteins, cGMP, Ca^2+^, calmodulin (CaM), and protein kinases may be second messengers in the GA signaling pathway (Thomas and Sun, 2004). In the cAMP signaling pathway system, an extracellular signal binds to the corresponding receptor and elicits a response by regulating the level of the intracellular second messenger cAMP (Kajana and Goshgarian, 2008). GA, a phytohormone, may act as a signaling molecule using adenylate cyclase for the regulation of cAMP levels, which, in turn, regulates key physiological processes. Zinc-finger transcription factors are among the major transcription factors in eukaryotes, of which Zn2Cys6-type zinc-finger transcription factors are considered to be fungal-specific transcription factors (MacPherson et al., 2006; Rohs et al., 2010). In *Chrysanthemum*, the zinc-finger transcription factor gene BBX24 is closely related to GA biosynthesis, and *BBX24* regulates flowering time and abiotic stress tolerance and thus affects normal growth and development of *Chrysanthemum* (Yang et al., 2014). In this study, we identified four upregulated zinc-finger transcription factors expressed during stipe elongation. The analysis revealed that v5-200357 (*FfZCP4*) and v5-210801 (*FfZCP68*) are both Zn2Cys6-type transcription factors, and v5-207229 and v5-212490 are both Cys2His2 (C2H2) type transcription factors. It is hypothesized that Cys2His2 (C2H2) and Cys6 (C6) zinc-finger type transcription factors have important roles in the process of GA affecting stipe elongation.

In our study, the expression of the β-1,6-glucan synthase gene *FfGs6* was significantly upregulated in yellow *F. filiformis*, which is consistent with the previous findings (Long et al., 2018). Expansin-like protein, a specific protein in the fungal cell wall, is involved in the formation of the cell wall structure (Sharova, 2007). Our study showed that *Expl2* expression was significantly upregulated during stipe elongation, and Expansin-like proteins have an important regulatory role for stipe elongation. *FfExg2* and *FfExg3* clustered together in the affinities for three different members of the exo-β-1,3-glucanase family of *F. filiformis*, and their expression patterns were similar, with *FfExg1* in different branches, with different expression patterns (Tao et al., 2018). It is consistent with our findings that *Exg1* gene expression was significantly upregulated, and *Exg2* and *Exg3* gene expression was significantly downregulated in the expression pattern.

**Figure 10:**
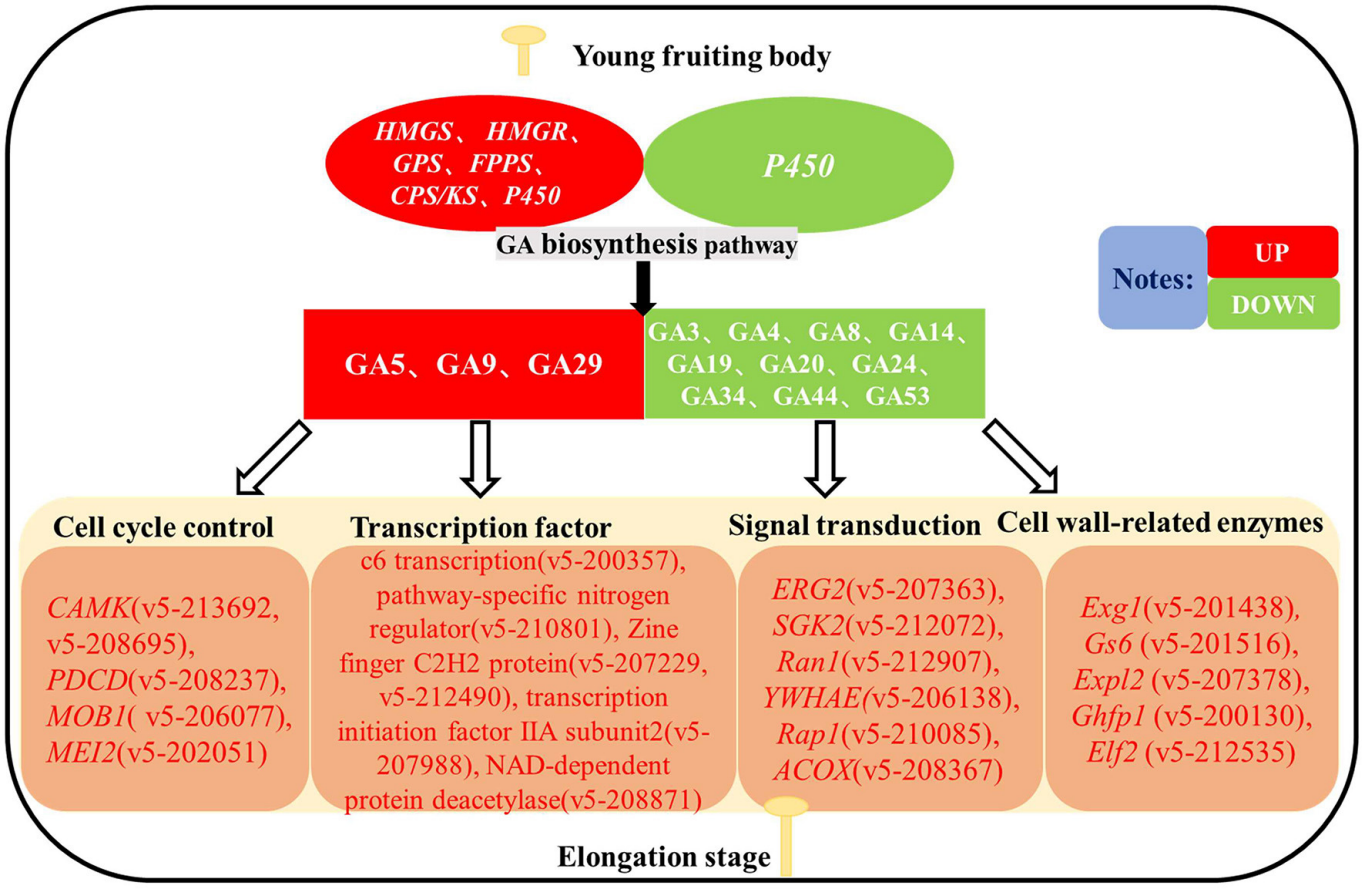
Hypothetical diagram of gibberellin regulating the stipe elongation of *F. filiformis*.

Based on the results of existing studies, we constructed a simulated molecular mechanism diagram of GA as an endogenous hormone regulating stipe elongation in *F. filiformis* (Figure 10). During the elongation of *F. filiformis*, differential expression of genes in the GA biosynthesis pathway causes changes in GA content, and GA acts on cell cycle control, transcription factor, signal transduction, and cell wall-related enzyme pathways to affect the synthesis of cell wall components and cell cycle changes in *F. filiformis*, thus regulating the stipe elongation, which is important for analyzing the molecular mechanism of GA regulation of stipe elongation in *F. filiformis*.

## Data availability statement

The datasets presented in this study can be found in online repositories. The names of the repository/repositories and accession number(s) can be found at: NCBI—PRJNA999472.

## Author contributions

HL, BX, and YT conceived and designed the research. HL, XM, WX, LS, and SY conducted the experiments. HL, SY, XM, HJ, ZL, YZ, BX, and YT analyzed the data and revised the manuscript. HL, SY, XM, and YT wrote the manuscript. All authors have read and approved the manuscript.
